# Dietary Intake and Cardiovascular Risk Factors in Icelanders Following Voluntarily a Low Carbohydrate Diet

**DOI:** 10.1371/journal.pone.0156655

**Published:** 2016-08-25

**Authors:** Anita S. Elidottir, Thorhallur I. Halldorsson, Ingibjörg Gunnarsdottir, Alfons Ramel

**Affiliations:** 1 Unit for Nutrition Research, Landspitali-The National University Hospital of Iceland and Faculty of Food Science and Nutrition, University of Iceland, Reykjavik, Iceland; 2 Centre for Fetal Programming, Statens Serum Institut, Copenhagen, Denmark; University College London, UNITED KINGDOM

## Abstract

**Background/Aim:**

Most studies regarding low-carbohydrate diets (LCDs) have been intervention studies. The aim of the current study was to investigate dietary intake and cardiovascular risk factors among individuals who voluntarily follow a LCD.

**Methods:**

A cross-sectional study was conducted (N = 54, 20–66yrs) in Reykjavik, Iceland. Participants recorded food intake for three days. Blood samples were analyzed for cardiovascular risk factors.

**Results:**

Nearly half of the participants were obese and around 60% had been on a LCD for ≥ 6 months. Fifty percent claimed they had lost weight during the past month. The median intake of carbohydrate, protein and fat were 8%, 22% and 68% E (hereof 25% saturated fatty acids), respectively. The consumption of bread and wholegrain cereals was very low (<5g/day), including the intake of dietary fiber (11g/day). Median fruit intake was 12 g/day. Intake of red meat and meat products was double that of the general population or ~900 g/week. Median intake of vitamins and minerals were mostly higher than the estimated average requirements. Cardiovascular risk factors were mostly within normal range. Mean blood lipids were slightly elevated although the high density lipoprotein/total cholesterol ratio was normal.

**Conclusion:**

Despite poor diet quality and high prevalence of obesity, individuals who voluntarily follow a LCD have cardiovascular risk factors mostly within reference range. These individuals consume very low amounts of carbohydrates and high amounts of fat and saturated fat acids. Intake of red meat and processed meat exceeds recommended intake. Very low intake of whole grain cereals and fruits results in low intake of fiber. Long term health implications need to be examined further in longitudinal studies.

## Introduction

Official dietary guidelines provide practical information for increasing the general level of public health and are based on the best available scientific evidence. These guidelines encourage adequate nutrition for health and body weight maintenance through dietary quality including appropriate intake of macro- and micronutrients [1, 2].

However, there are dietary regimens, i.e., low-carbohydrate diets (LCDs) which have become increasingly common during the last few decades, gaining a particular boost in popularity during the 1970s, when the Atkins Diet became widely known. Definition of LCD as provided by Atkins includes greatly restricting carbohydrates with increased protein- as well as increased total fat and saturated fatty acid intake [3]. Currently there is no consensus on the definition of a LCD and the energy from carbohydrates in available studies ranges from 4–45% E [4–8]. They all embrace a carbohydrate intake lower than recommended resulting in severe restrictions of carbohydrate-containing food groups that are generally considered part of a healthy diet, such as whole grain cereals, dairy products, legumes, fruits and root vegetables. Therefore, this type of diet is not in agreement with dietary recommendations [1].

Assessing the potential health consequences of LCDs is, however, complicated by the fact that LCDs appear to be useful for weight loss with several, studies showing that LCDs can lead to even greater weight loss than low fat diets (LFDs) [9–14], although not all studies agree [4, 15–18]. Surprisingly, many interventions have suggested that LCDs do not have adverse effects on cardiovascular risk factors despite a high intake of saturated fats [10, 13, 19–21]. In this context it is relevant to keep in mind that the weight loss associated with LCD diets may, on its own, lead to favorable changes in cardiovascular risk factors. Thus from previous studies it has been difficult to separate the independent effects of the LCDs from that of weight loss on cardiovascular risk [22–24].

Most of the studies on LCDs have focused on total energy intake and macronutrient distribution rather than dietary quality, e.g., intake of food groups that are of importance for health and disease. Additionally, only a limited number of studies have studied micronutrient intake related to LCDs, which indicate that individuals who follow a LCD commonly do not reach the recommended intake for several micronutrients, e.g., vitamin C, folic acid and iron [25–27]. Furthermore, according to the best of our knowledge, there are no studies available that investigate dietary habits among subjects that claim to habitually follow a LCD as currently available studies are limited to intervention studies. It is worth considering the dietary intake of those who voluntarily follow LCD because their food selection may differ from a prescribed diet used in intervention studies.

In order to gain knowledge on dietary intake and cardiovascular risk in individuals who voluntarily follow a LCD, we conducted this cross-sectional study. The aims were to examine 1) the dietary intake in this group for both macro–and micronutrients; 2) the consumptions of major food groups; and 3) cardiovascular risk factors in this group.

## Methods and Materials

### Subjects and study design

This was a cross-sectional study which investigated Icelandic individuals who voluntarily follow a LCD. All participants (N = 54) were from the capital area of Iceland and were recruited through an advertisements on social media groups dedicated to low carbohydrate diet. Those who responded to the advertisement were included in the study if, on participants' self-report, they were currently following voluntarily a LCD. Exclusion criteria were pregnancy and/or breastfeeding. Permission for this study was granted from the Icelandic National Bioethics Committee (VSNb2014060024/03.07). All participants gave their written informed consent prior to their inclusion in the study.

### Dietary assessment

Diet was assessed using a 3-day weighted food record. Participants weighed and recorded their food intake for three consecutive days, two week days and one weekend day. Instructions on how to record the diet were given. The results of the food records were typed into the food calculation program ICEFOOD 2.0 based on the ISGEM databank (www.matis.is), which contains data on the composition of foods on the Icelandic market. The nutritional data from ISGEM anticipates loss of certain nutrients during different cooking methods. Energy is calculated as: 9 kcal/g for fats, 7 kcal/g for alcohol, 4 kcal/g for protein and carbohydrates and 2 kcal/g for fiber.

### Anthropometric measurements

Height was measured with a calibrated stadiometer (model no. 206; Seca, Hamburg, Germany). Body weight and body composition were measured in light underwear using a bioelectrical impedance analysis device (InBody230, InBody, Seoul, Korea). Body mass index (BMI) was calculated from the recorded height and weight (kg/m^2^). Waist circumference was measured halfway between the top of the lateral iliac crest and the lowest rib.

### Blood samples

Participants were instructed to avoid strenuous exercise and alcohol consumption the day before the drawing of fasting blood samples. The blood was collected within 3 days of completion of the 3-day weighed food record. The blood samples were centrifuged and the serum was stored at -80°C for subsequent analyses. Blood analyses were conducted at the central laboratory of Landspitali–The University Hospital in Reykjavik, Iceland. Reference values were used as suggested by the laboratory. Total cholesterol (TC), plasma triglycerides (TG) and glucose were analyzed using an enzymatic colorimetric assay and an automated analyzer (Hitachi 911; Roche Diagnostics, Indianapolis, USA). High density lipoprotein (HDL) was determined using polyethylene glycol-modified enzymes and dextran sulfate. Low density lipoprotein (LDL) was estimated using the Friedewald formula. Insulin was measured with electro-chemo-luminescence immunoassay on a Modular Analytics E170 system from Roche Diagnostics (Manheim, Germany).

### Questionnaires

We used the web-based survey tool LimeSurvey (https://www.limeservice.com/en/) to ask participants questions about socioeconomic status, health related behavior, e.g., smoking habits, alcohol consumption, health status (food allergy or intolerance), usage of medication, questions about physical activity and intake of dietary supplements.

### Statistical analyses

Statistical analyses were performed using the IBM SPSS statistical package, version 22.0 (SPSS, Chicago, IL, USA), and all variables were examined for normality using histograms and QQ-plots. Continuous variables were presented both as means ± standard deviations (SD); as well as median and interquartile range (IQR). Chi-square test was used to test differences in categorical variables between two groups. An Independent-Samples T-test was used to assess significant differences in continuous variables between two groups when distributions were normal and Mann-Whitney test was used for skewed distributions. One-Sample-T-test was used to calculate whether dietary intake of the participants was different from the Nordic Nutrition Recommendations 2012 or from the mean intake reported from the Icelandic Nutrition Survey 2010/2011 when distributions were normal and One-Sample-Mann-Whitney test was used for skewed distributions. Correlations between continuous variables were estimated using Spearman's rho correlation coefficient. According to power calculations the sample size was sufficient to detect a significant difference in protein intake by 2% compared to mean intake from the Icelandic Nutrition Survey 2010/2011. Similar numbers were received for total fat (3%), fatty acids (2%) and carbohydrates (3%). The level of significance was set at p = 0.05.

## Results and Discussion

Fifty-four subjects participated in the study. Table 1 shows their characteristics. More than two-thirds of the participants were women. According to BMI, 87% of participants were at least overweight (>25 kg/m^2^) and 47% were obese (>30 kg/m^2^). The majority of the participants reported being on a LCD ≥ 6 months. Only two subjects used blood lipid drugs and one subject used anti- diabetic drugs.

**Table 1:** Characteristics of the participants.

	All subjects			Female			Male		
	n = 54			n = 38			n = 16		
	Mean	±	SD	Mean	±	SD	Mean	±	SD
Age (years)	43	±	11	43	±	11	43	±	11
Height (cm)	169	±	8	165	±	6	178	±	6
Body weight (kg)	85	±	13	83	±	13	90	±	10
Waist circumference (cm)	97	±	12	96	±	14	99	±	8
BMI (kg/m^2^)	30	±	4	30	±	4	28	±	3
Body fat (%)	34	±	12	40	±	7	21	±	9
Fat free mass (kg)	56	±	12	49	±	5	71	±	11
Smoking ever (yes in %)		51.9			52.6			50.0	
Weight loss during the last month (%)		50.0			55.3			37.5	
≥ 6 months on LCD (%)		59			47			88*	

Data are shown as mean ± standard deviation (SD) and as percentage in yes.

*Significant difference between men and women

Table 2 shows the consumption of major food groups according to the 3-days food records, along with results from the Icelandic National Nutrition Study 2010/2011 for comparison. Consumption of fruits, bread, wholegrain cereals, potatoes, rice and pasta was very low, while consumption of meat and meat products was high. There was almost no consumption of sugar, sweets and soft drinks, but a rather high intake of sugar-free soft drinks.

**Table 2:** Daily consumption (g) of food groups in comparison to the Icelandic nutrition survey from 2010/2011.

	All subjects (N = 54) percentiles						Icelandic Nutrition Survey 2010/2011* (N = 1312)		
*Food groups in g/day*	Mean	±	SD	25	50	75	Mean	±	SD
Vegetables^1^	171	±	93	116	149	238	120	±	100
Fruits^2^	38	±	61	0	12	54	119	±	120
Bread and crisp bread^2^	36	±	36	6	25	61	95	±	61
Wholegrain cereals^2^	4	±	11	0	0	2	22	±	33
Meat and meat products^2^	131	±	82	64	121	177	70	±	83
Fish and seafood	45	±	52	7	33	59	46	±	62
Poultry^1^	62	±	65	0	47	105	27	±	47
Solid fats**	14	±	12	5	12	20	12	±	14
Oils***^2^	8	±	11	0	4	13	2	±	5
Milk and dairy products^2^	206	±	179	72	181	291	300	±	232
Cheese^1^	47	±	38	20	37	71	35	±	31
Eggs^1^	72	±	58	33	56	102	12	±	23
Sugar, honey and candy	8	±	19	0	0	5	17	±	28
Soft drinks with sugar^2^	0	±	0	0	0	0	127	±	249
Sugar-free soft drinks^1^	225	±	389	0	0	333	74	±	229

Data are displayed as mean results and ± standard deviation (SD) and as percentiles.

* Thorgeirsdottir H et al. [What do Icelanders eat? National dietary survey 2010–2011]. The Iceland Health Directorate 2011, Reykjavik, Iceland.

**Includes all animal fats (except fish), butter, margarine and hydrogenated fats

*** Includes all unhydrogenated vegetable oils

^1^ significantly higher intake than mean intake reported from the Icelandic Nutrition Survey 2010/2011

^2^ significantly lower intake than mean intake reported from the Icelandic Nutrition Survey 2010/2011

Table 3 shows the intake of energy and macronutrients composition, together with the recommended daily intake according to the Nordic Nutrient Recommendations 2012. The median for total energy intake was 1800 kcal, with an expected difference between women and men (1750 vs. 2030 kcal, P = 0.018, respectively). The intake of macronutrients was significantly different (P < 0.001) from the intake reported in the latest Icelandic National Survey, i.e., lower for carbohydrates (10 vs. 42%) and dietary fiber (12 vs. 17 g), but higher for protein (23 vs. 18%), total fat (66 vs 36%), saturated—(26 vs. 15%), monounsaturated—(21 vs. 12%) and polyunsaturated fatty acids (12 vs. 6%). The distribution of macronutient intake as %E was similar between the two sexes.

**Table 3:** Daily intake of energy and energy giving nutrients compared to recommended intake.

				Percentiles			Recommendeddaily intake**
	Mean		SD	25	50	75	
Energy (kcal)	1928	±	534	1590	1836	2155	
Protein (%E)^1^	23	±	5	19	22	27	10–20
Fat (%E)^1^	66	±	8	63	68	71	30–40
Saturated f.a.* (%E)^1^	26	±	6	21	25	32	< 10
Monounsaturated f.a.* (%E)	21	±	3	19	21	23	10–20
Polyunsaturated f.a.* (%E)^1^	12	±	4	10	11	13	5–10
Carbohydrates (%E)^2^	10	±	6	6	8	11	45–60
Fiber, total (g)^2^	12	±	7	7	11	16	25–35

Data is displayed as mean results and ± standard deviation (SD) and as percentiles

* f.a. = fatty acids

** Nordic Nutrition Recommendations 2012.

^1^ significantly higher intake than recommended

^2^ significantly lower intake than recommended

Table 4 shows an overview of vitamins and minerals intake. The median intake of vitamins and minerals was mostly higher than the estimated average requirements. Intake of sodium (P = 0.003) and vitamin B_12_ (P < 0.001) was significantly higher than recommended, women's intake of iron (P < 0.001) was significantly lower than recommended.

**Table 4:** Daily micronutrient intake among male and female participants.

	Females (n = 38)			Males (n = 16)				
		Percentiles			Percentiles		Recommended	Average
	25	50	75	25	50	75	daily intake	requirement
Vitamin A (μg)	655	880	1127	687	956	1460	700 / 900*	500/600
Vitamin D (μg)	5	8	16	5	14	27	15	7.5
Vitamin E (mg)	13	17	21	15	16	28	8 / 10*	6
Thiamin (mg)	0.8	1.0	2.1	1.1	1.6	2.5	1.1 / 1.3*	1.2
Folate (μg)	182	275	517	202	319	497	300	200
Vitamin B_12_ (μg)^1^	5	7	9	6	7	11	2	1.4
Vitamin C (mg)	30	76	122	29	53	106	75	60
Calcium (mg)	500	774	1016	486	770	1050	800	500
Sodium (mg)^1^	1823	2560	3352	2479	2837	3832	2400	-
Iron (mg)^2^	6	9	12	5	11	12	9 /15*	7
Phosphorus (mg)	1298	1418	1682	1424	1750	1962	600	450
Magnesium (mg)	200	254	314	198	245	346	280/350*	-

* Recommended intake differs between sexes, lower numbers are for women and higher numbers are for men.

^1^ significantly higher intake than recommended

^2^ significantly lower intake in women than recommended

Table 5 shows biomarkers of cardiovascular disease along with reference values. Total cholesterol, HDL and LDL were high and a considerable percentage of participants exceeded the corresponding reference values. The total cholesterol/HDL cholesterol ratio was well within normal range. The mean of glucose was within normal range and only few exceeded the limits for triglycerides and insulin. Cardiovascular risk factors were not associated with self-reported length of LCD or previous weight loss (data not shown), however, fasting blood glucose correlated with energy% from carbohydrates (Spearman's rho = 0.282, P = 0.039). As expected, women had higher HDL than men (1.7 ± 0.6 vs. 1.3 ± 0.5, P = 0.025) and obese participants had higher fasting insulin than normal- and overweight participants (10.3 ± 5.1 vs. 5.9 ± 3.8 mU/L, P = 0.001).

**Table 5:** Cardiovascular risk factors measured in fasting blood samples.

	Mean	±	SD	% above reference value	reference values
Blood glucose (mmol/L)	5.3	±	0.6	37%	< 5.5
Total cholesterol (mmol/L)	5.2	±	1.7	50%	< 5.0
LDL (mmol/L)	3.1	±	1.3^1^	61%	< 2.6
HDL (mmol/L)	1.6	±	0.6^1^	26%	> 1.1
Triglyceride (mmol/L)	0.9	±	0.4	7%	< 1.7
Insulin (mU/L)	7.6	±	4.8	0%	2.6–24.9

Data is shown as mean ± standard deviation (SD) and as percentage of subject outside reference.

^1^ significantly higher intake than reference value

This cross-sectional study investigated Icelandic individuals who voluntarily followed a LCD. According to our results, these individuals consumed very low amounts of carbohydrates and very high amounts of fat. According to anthropometric measurements, this group was not in good physical condition displaying high body fatness, however, cardiovascular risk factors measured in blood were mostly within normal range.

The majority of our participants reported being on a LCD for ≥ 6 months. However, their lifestyle and physical condition were far from optimal. Out of all the participants, 50% smoked and only half of the participants were physically active at least 30 minutes a day. According to anthropometric measurements 19% of men and 46% of women had a BMI > 30 kg/m^2^ and 19% of men and 71% of women had high waist circumference above 102/88 cm [28].

Considering that the subjects followed a LCD voluntarily and were not part of a dietary intervention prescribing strict carbohydrate restriction, the carbohydrate intake of this group was surprisingly low (8% of energy intake from carbohydrates), which was achieved by little to no consumption of cereals, limited intake of low fat dairy products, and low consumption of fruits. Thus, this group followed a LCD as defined by Atkins [3] and consequently not a LCD as defined by others as summarized in a recent systematic review and meta-analysis by Hu et al (2012) that interprets a LCD as maximum energy intake of 45 percent from carbohydrates. [4].

Given the high prevalence of overweight/obesity, low physical activity and very high fat intake among our study participants, an unfavorable risk profile for biomarkers of cardiovascular health would be expected. Around 50% of the study participants had total cholesterol above the desirable limits [29]. Further on, our results also suggest that LDL cholesterol was considerably high, with 61% of the subjects over the desirable limit (2.6 mmol/L) [29]. It is well known that foods rich in saturated fatty acids increase total- and LDL cholesterol [30, 31] but it is also suggested that a diet rich in saturated fatty acids increases HDL and decreases triglycerides [31, 32]. Consequently, our results show that 74% of the participants had HDL concentrations above the desirable value of 1.1 mmol/L and only 7% of the participants exceeded the upper limits for circulating triglycerides [29]. Fasting blood glucose was in the normal range for the majority of participants (78% below 5.5 mmol/L) and correlated with energy% from carbohydrates. It has been previously reported that LCD can be effective for normalizing blood glucose [13] and the low carbohydrate intake in the present study might have helped our participants to keep fasting blood glucose within acceptable limits despite physical inactivity and obesity. No participant was above the reference value for fasting insulin, which is perhaps not surprising given the low consumption of carbohydrates [33]. The observed levels of these above mentioned biomarkers were not higher, and actually quite comparable to more representative sample from the Icelandic population [34–35].

In this context it is important to ask how detrimental the blood values in our participants were in relation to cardiovascular risk. Although both total cholesterol and LDL are considered atherogenic and were high in the subjects, HDL levels were also high thus resulting in a favorable ratio between total cholesterol and HDL of only 3.3 (a lower ratio is considered better regarding cardiovascular risk with a reference value below 4) [36]. Most importantly, the total/HDL ratio is an indicator of vascular risk and its predictive value is greater than that of single blood lipid parameters [36]. However, it might still be beneficial for our individuals to place more emphasis on polyunsaturated fatty acid intake which have been shown to reduce risk for several diseases [30, 31, 37].

In comparison to the dietary intake of the general Icelandic population, our participants consumed more red meat and processed meat [38]. Guidelines recommend a consumption of less than 500 grams of red meat per week, or ~70 g/day [1], whereas consumption in our study participants was on average 130 g/day (900 grams per week). Our participants also consumed more butter, oils, sauces, mayonnaise-based salads and eggs, although dairy product intake was lower than the Iceland average. Consequently, this dietary pattern reflects high intake of protein and fat. Although the vegetable intake was below the recommended intake among our study participants, it was still higher than the intake in the latest Icelandic Nutrition Survey. Given the potential beneficial effects of vegetables on CHD risk [39] it is possible that this relatively higher vegetable intake may counteract the potential adverse effect of high fat intake on CVD risk factors in this population.

Taking into consideration the subjects' consumption of cereals and fruits, fiber intake was inevitably low. It has been suggested that when carbohydrates are severely limited for a long time period this can result in insufficient intake of folic acid, thiamine, vitamin C, calcium, iron and magnesium [14, 25, 26]. In our study median intake of these nutrients mostly reached average requirements and thus our results are not in accordance with the aforementioned studies.

### Strengths and limitations

It is the strength of this cross sectional survey that it provides insight into food selection, nutrient intake and associated cardiovascular risk among participants who voluntarily follow a LCD. It therefore gives realistic information about dietary habits of LCD follower, in contrast to dietary habits recorded in intervention settings. Although we cannot exclude the possibility that previous weight loss affected cardiovascular risk in our subjects, we did not observe any difference between CVD risk in subjects that experienced weight loss and subjects that did not.

A limitation of this study is that it was of cross sectional design so we were not able to differentiate between cause and consequence when examining biomarkers of health. It should also be stated that the accuracy of a food diary is highly dependent on the level of care and attention shown by the participants when recording dietary intake [40]. Therefore, as with all dietary assessment methods, accuracy in reporting in terms of over and underreporting is problematic. As a result we cannot exclude that dietary habits were in some cases more extreme than usual for some of the participants being asked to record their diet. Despite the possibility of such bias, the recorded diet should in most cases reflect the habits or intentions of participants trying to follow a low carbohydrate diet and our results should be interpreted in that context.

## Conclusions

Despite poor diet quality, low physical activity and high prevalence of obesity, individuals who voluntarily follow a LCD have cardiovascular risk factors mostly in reference range. These individuals consume very low amounts of carbohydrates and high amounts of fat and saturated fat acids. Intake of red meat and processed meat exceeds recommended intake. Very low intake of whole grain cereals and fruits results into a low intake of fiber. Long term effects need to be determined.

## Abbreviations

LCD: Low-carbohydrate diet
LFD: Low fat diet
BMI: Body mass index
TC: Total cholesterol
TG: Plasma triglycerides
HDL: High density lipoprotein
LDL: Low density lipoprotein
IQR: Interquartile range
SD: Standard deviation
